# Complete genome sequence of PETase type IIa-harboring *Marinobacter nanhaiticus* D15-8W, isolated from a South China Sea sediment

**DOI:** 10.1128/MRA.00868-23

**Published:** 2023-11-09

**Authors:** Yukako Suzuki, Ayako Fukazawa, Koki Sugawara, Josephine Galipon, Kazuharu Arakawa

**Affiliations:** 1 Institute for Advanced Biosciences, Keio University, Tsuruoka, Yamagata, Japan; 2 Systems Biology Program, Graduate School of Media and Governance, Keio University, Fujisawa, Kanagawa, Japan; 3 MIRAI Technology Institute, Shiseido Co., Ltd., Yokohama, Kanagawa, Japan; 4 Yamagata Prefectural Sakata Higashi High School, Sakata, Yamagata, Japan; 5 Graduate School of Science and Engineering, Yamagata University, Yonezawa, Yamagata, Japan; 6 Faculty of Environment and Information Studies, Keio University, Fujisawa, Kanagawa, Japan; California State University San Marcos, San Marcos, California, USA

**Keywords:** *Marinobacter*, halophile, PETase, bioremediation

## Abstract

*Marinobacter nanhaiticus* D15-8W is known for its ability to metabolize polycyclic aromatic hydrocarbons. Here, we report the complete circular genome sequence of this strain to be 5,336,660 bp (G + C content, 58.6%; 4,869 protein-coding sequences) with one plasmid (69,655 bp).

## ANNOUNCEMENT

*Marinobacter nanhaiticus* D15-8W is a slightly halophilic bacilliform Gram-negative bacteria that is facultatively anaerobic and was previously isolated from a South China Sea sediment (1). The ability of some *Marinobacter* strains, including *M. nanhaiticus* D15-8W, to degrade polycyclic aromatic hydrocarbons has been a major focus of past research (1–5). In addition, *M. nanhaiticus* D15-8W is currently one of the few known marine bacteria to harbor a gene with the potential to degrade poly(ethylene terephthalate) (PET), characterized as PETase type IIa (6). The draft genome sequence of this strain, consisting of 14 contigs, was published previously (7), but here we report the complete circular genome for this strain.

*M. nanhaiticus* D15-8W strain was purchased from the Korean Collection for Type Cultures as KCTC 23749^T^. After streaking on Marine Agar 2216, a single colony was cultured overnight in Marine Broth 2216 (product no. 76448, MilliporeSigma; 30°C, with shaking at 200 rpm) and harvested at an OD_600_ of around 0.3. The genomic DNA was extracted using the Genomic-tip 20/G system (Qiagen). A genomic DNA library for sequencing was prepared and multiplexed using the rapid barcoding kit (product no. SQK-RBK004, Oxford Nanopore Technologies). The library was sequenced with FLO-MIN111 flow cell (Oxford Nanopore Technologies) on a GridION X5 device (GridION software version 21.10.5, Oxford Nanopore Technologies), base-called, demultiplexed, and adapter-trimmed by guppy version 6.3.8 (Super-Accurate Mode). Illumina sequencing was performed for error correction using the HyperPlus library preparation kit (Kapa Biosystems) and a MiSeq sequencer with Reagent Kit v3 600 cycles as 300 bp paired ends (Illumina). All nanopore reads (31,320 reads, 126 Mbp, N50 length 7.5 kbp) were used for the *de novo* assembly using Canu version 2.2 (8). The resulting single contigs of a chromosome and a plasmid were circularized by manually deleting the overlapping ends. An improved consensus sequence was obtained by correcting the draft assembly with all raw Illumina reads (8.6M pairs) using the Pilon software version 1.23 (9). The resulting genome sequence was functionally annotated using DFAST version 1.2.18 (10). The assembled genome consists of one circular chromosome of 5,336,660 bp with 58.6% G + C content, including 4,869 coding sequences (CDSs), 52 tRNAs, and 9 rRNAs. Moreover, one plasmid was found (length, 69,655 bp; G + C content, 54.3%; CDSs, 80), on which oriTfinder detected nine essential genes from the type IV secretion system (11). Default parameters were used except where otherwise noted.

The assembly completeness was assessed with CheckM (DFAST), resulting in 99.61% completeness with the Alteromonadaceae marker lineage (12), which was equivalent to the previously published draft assembly (7). We confirmed that the newly sequenced genome contains the gene for PETase type IIa, as reported previously, by BLASTP search of UniProt A0A0K8P6T7 PETase (*e* = 3*e*−75) (6). The complete genome reported here may contribute to understanding how *M. nanhaiticus* D15-8W decomposes organic pollutants in marine environments.

## Data Availability

The genome sequences reported here were deposited in DDBJ under accession numbers AP028878 and AP028879, and the raw reads were deposited in the Sequence Read Archive (SRA) under BioProject accession number PRJNA1011029 as SRR25824389 and SRR25824390 runs.
